# The Mechanism of the Solute-Free Water Reabsorption Increase in the Rat Kidney by Oxytocin Saluresis

**DOI:** 10.1134/S1607672921020113

**Published:** 2021-03-05

**Authors:** Yu. V. Natochin, E. I. Shakhmatova, A. E. Bogolepova

**Affiliations:** Sechenov Institute of Evolutionary Physiology and Biochemistry, Russian Academy of Sciences, St. Petersburg, Russia

**Keywords:** kidney, oxytocin, vasopressin, reabsorption of solute-free water, saluresis

## Abstract

We found an experimental solution to the paradox when the reabsorption of solute-free water increases with a simultaneous increase in diuresis and saluresis in the rat kidney under the oxytocin action. Injection of oxytocin to rats (0.25 nmol/100 g of body weight) increases diuresis from 0.16 ± 0.03 to 0.26 ± 0.02 mL/h, the excretion of solutes from 134 ± 13.7 to 300 ± 16.3 μOsm/h, and the reabsorption of solute-free water, which correlates with the renal excretion of oxytocin (*p* < 0.001). The mechanism of the effect is that oxytocin decreases the reabsorption of ultrafiltrate in the proximal tubule (the clearance of lithium increases) and increases the fluid flow through the distal segment of the nephron. In vivarium rats, urine osmolality (1010 ± 137 mOsm/kg H_2_O) and the concentration of vasopressin are high, this causes an increase in the reabsorption of solute-free water. Thus, oxytocin increases saluresis, which, against the background of a high level of endogenous vasopressin, increases the water reabsorption in the collecting ducts.

Neurohypophysial nonapeptides (oxytocin and vasopressin) affect the renal excretion of sodium and water by the kidney [1–3]. Oxytocin causes natriuresis and increases urine excretion. It was found that vasopressin can induce natriuresis and increase the reabsorption of solute-free water in the mammalian kidney. These effects are due to the stimulation of different subtypes of V receptors [2, 4, 5]. In contrast to vasopressin, which has three subtypes of these receptors, only one type of oxytocin receptor gene was found in the rat genome [6]. Two assumptions have been made about the molecular mechanism of action of oxytocin on the reabsorption of water and sodium in the kidney. According to one of them, it stimulates vasopressin V_2_ receptors and increases water reabsorption [7], and an increase in sodium excretion is explained by the formation of new forms of oxytocin (OT-GKR) [8]. Our hypothesis is that the effect of oxytocin on water reabsorption under the influence of physiological concentrations of this hormone can be explained without assuming the stimulation of receptors of other nonapeptides. The objective of this study was to experimentally substantiate the new hypothesis of the physiological mechanism of oxytocin action basing on the classical concepts of the renal function.

Experiments were performed on female Wistar rats (*Rattus norvegicus* var. *albino*) weighing 200–250 g. The animals received standard granulated food (recipe PK-120, Aller Petfood LLC, Russia) and had free access to water. In the morning of the day of experiment, the rats were not fed. During the experiment, the rats were placed in individual cage-cases with a wire bottom, through which the urine flowed down a funnel into a measuring tube. Diuresis was recorded during spontaneous urination for 2 h. The number of animals in each series is indicated in the captions to Figs. 1-4 and Table 1. The osmolality of blood serum and urine samples was determined cryoscopically with an Osmo1 microosmometer (Advanced Instruments, United States). The concentration of sodium and potassium in urine samples was measured with a Sherwood-420 flame photometer (Sherwood Scientific, Great Britain), and the concentration of these ions in blood serum was determined using an Erba XL-200 automatic biochemical analyzer with an ion-selective unit (Lachema, Czech Republic). The concentration of lithium in urine was determined in an air-acetylene flame with an AA-6200 atomic absorptiometer (Shimadzu, Japan), and the concentration of lithium in blood serum was determined with a Sherwood-420 flame photometer. The concentration of oxytocin and arginine-vasopressin (AVP) in urine samples was determined using enzyme-linked immunosorbent assay kits (Enzo Life Sciences, United States). The optical density was measured in 96-well plates according to the test system method with an ELx808 automatic reader (Bio-Tek Instruments, United States). Lithium clearance was used as a marker of proximal fluid reabsorption. In the vivarium, rats received lithium chloride for 7 days before the experiment according to the following scheme: in the first 3 days, the water in the drinkers was replaced with a LiCl solution (1.5 (mg/100 g per day)); in the next 4 days, a LiCl solution was injected daily at a dose of 0.5 mg/(100 g of body weight per day). In the experiments, we used oxytocin (Sigma-Aldrich, United States) and LiCl (NevaReaktiv, Russia). Renal function parameters were calculated using the standard formulas per 100 g of animal body weight. The clearance of solute-free water (${\text{T}}_{{{{{\text{H}}}_{2}}{\text{O}}}}^{{\text{C}}}$) was calculated by the formula ${\text{T}}_{{{{{\text{H}}}_{2}}{\text{O}}}}^{{\text{C}}}$ = C_Osm_ – V, where С_Osm_ is clearance from osmotically active substances, and V is diuresis. C_Osm_ is calculated from serum and urine osmolality data. All data are presented as *M* ± *m*. Comparison between groups was performed using one- or two-way analysis of variance and Holm–Sidak multiple comparison test, *r* is Pearson’s correlation coefficient. Differences were considered statistically significant at *p* < 0.05.

**Table 1.** Parameters of the osmoregulatory function of the kidney and the excretion of nonapeptides within the first hour after the oxytocin injection

Kidney function parameters	Control, 0.9% NaCl (*n* = 8)	OT 0.25 nM (*n* = 15)
V, mL/h	0.16 ± 0.03	0.26 ± 0.02^#^
U_Osm_, mOsm/kg H_2_O	1010 ± 137	1284 ± 89^NS^
U_Na_, μmol/mL	65.0 ± 18.3	270.1 ± 31.2^##^
U_K_, μmol/mL	109.0 ± 23.5	102.9 ± 9.9^NS^
U_Osm_V, μOsm/h	134.0 ± 13.7	300.3 ± 16.3^##^
U_Na_V, μmol/h	7.8 ± 1.8	64.6 ± 8.4^##^
U_K_V, μmol/h	13.3 ± 2.6	24.3 ± 2.5^#^
${\text{T}}_{{{{{\text{H}}}_{2}}{\text{O}}}}^{{\text{C}}}$, mL/h	0.28 ± 0.04	0.75 ± 0.04^##^
U_ОТ_V, pg/h	175.0 ± 26.8	1598 ± 233^##^
U_AVP_V, pg/h	23.0 ± 4.8	29.3 ± 3.1^NS^

Oxytocin was injected at a dose of 0.25 nM/(100 g of body weight). Designations: U_Osm_, U_Na_, U_K_—concentration of osmotically active substances, sodium, and potassium in urine, respectively; V, U_Osm_V, U_Na_V, U_K_V, ${\text{T}}_{{{{{\text{H}}}_{2}}{\text{O}}}}^{{\text{C}}}$, U_OT_V, U_AVP_V—diuresis, excretion of osmotically active substances, sodium, and potassium, reabsorption of solute-free water, and excretion of oxytocin and AVP, respectively.

Significance of differences from the control: ^#^ *p* < 0.02; ^##^ *p* < 0.001; ^NS^ *p* > 0.05.

Oxytocin injection at a dose of 0.25 nM/(100 g of body weight) increases renal sodium excretion. Studies have shown that, in the dose range from 0.175 to 0.325 nM/(100 g of body weight), oxytocin had almost the same saluretic effect, which made it possible to use a dose of 0.25 nM/(100 g of body weight) to study the effect of oxytocin on the renal excretion of water and solutes. To elucidate the mechanism of the saluretic effect of oxytocin, we studied the ratio of the excretion of osmotically active substances and the clearance of lithium, which characterizes the reabsorption of fluid in the proximal segment of the nephron. Oxytocin injection reduces fluid reabsorption in the proximal nephron segment, and lithium clearance and renal excretion of osmotically active substances simultaneously increase (Fig. 1). This is observed at a stable level of glomerular filtration: creatinine clearance (*С*_Cr_) is 0.45 ± 0.03 mL/min in the control and 0.46 ± 0.03 mL/min after the injection of oxytocin. The excretion of oxytocin with the release of osmotically active substances (Fig. 2). The increase in diuresis was due to the release of sodium ions, which correlates with an increase in the lithium clearance (Fig. 3), with a simultaneous increase in the reabsorption of solute-free water (Fig. 4). The experiments were performed on rats from the vivarium, which were normally in a state of antidiuresis and had a high level of endogenous vasopressin in blood: urine osmolality exceeded 1000  mOsm/kg H_2_O (Table 1). The oxytocin-induced flow of a larger volume of tubular fluid passes through the renal collecting ducts, the osmotic permeability of the wall of which is increased due to the presence of vasopressin in blood.

**Fig. 1.**
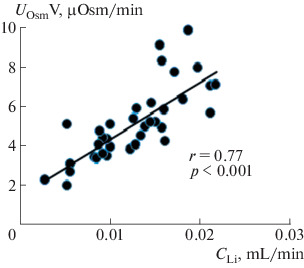
Correlation between the excretion of osmotically active substances and renal clearance of lithium in rats after oxytocin injection. The abscissa axis shows lithium clearance (*С*_Li_), and the ordinate axis shows the excretion of osmotically active substances (*U*_Osm_V) after the injection of 0.25 nM oxytocin (*n* = 15).

**Fig. 2.**
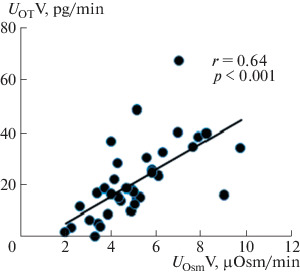
Correlation between the excretion of osmotically active substances and oxytocin by the rat kidney. The abscissa axis shows the excretion of osmotically active substances (*U*_Osm_V) after the injection of 0.25 nM oxytocin (*n* = 15), and the ordinate axis shows the excretion of oxytocin (*U*_OT_V).

**Fig. 3.**
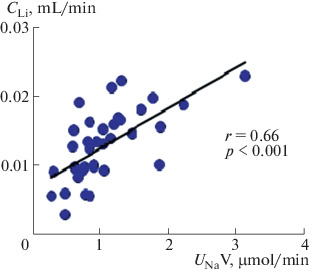
Correlation between the sodium excretion and renal clearance of lithium in rats after oxytocin injection. The abscissa axis shows the sodium excretion (*U*_Na_V) after injection of 0.25 nM oxytocin (*n* = 15), and the ordinate shows lithium clearance (*C*_Li_).

**Fig. 4.**
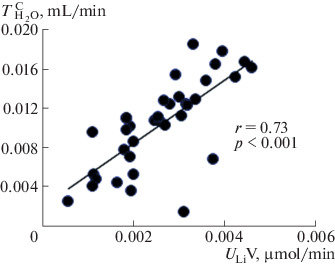
Correlation between the excretion of lithium and reabsorption of solute-free water in the rat kidney after the oxytocin injection. The abscissa axis shows the lithium excretion (*U*_Li_V), and the ordinate axis shows the reabsorption of solute-free water (${\text{T}}_{{{{{\text{H}}}_{2}}{\text{O}}}}^{{\text{C}}}$) after the injection of 0.25 nM oxytocin (*n* = 15).

The increase in saluresis and diuresis under these conditions increased the solute-free water reabsorption (Fig. 4). This was due to the fact that, at significant concentrations of vasopressin, this hormone stimulates V_2_ receptors of the collecting duct cells_._ Against the background of a decrease in proximal reabsorption under the influence of oxytocin and at a significant concentration of endogenous vasopressin, the increase in the fluid flow through the tubule is accompanied by an increase in the solute-free water reabsorption (Fig. 4, Table 1).

The study of the mechanism of the physiological effect of oxytocin on the renal function was performed on non-anesthetized animals, under standard conditions in relation to the state of the renal function. A physiological paradox was revealed: the antidiuretic renal response (increased reabsorption of solute-free water) at a stable glomerular filtration (constant creatinine clearance) was combined with an increase in diuresis in response to the oxytocin injection. The physiological mechanism of this effect of oxytocin in rats can be explained as follows. Oxytocin stimulates OT receptors in the cells of the proximal nephron segment. This decreases the proximal fluid reabsorption and is confirmed by an increase in lithium clearance. This is accompanied by an increase in the inflow of fluid into the distal segment of the nephron, in which oxytocin at the dose used stimulates V_1a_ receptors. Blockade of these receptors by a selective V_1a_ receptor antagonist eliminates the natriuretic effect of oxytocin [9]. As a result, the reabsorption of ions in the thick ascending part of the Henle loop decreases, which is accompanied by an increase in the excretion of osmotically active substances, in saluresis and diuresis, and, simultaneously, in the reabsorption of solute-free water (Table 1). The first two effects depend on the injected oxytocin, and the last effect is determined by the high level of endogenous vasopressin, because the experiment was performed on the standard animals from the vivarium, in which the level of the endogenous hormone in blood and, accordingly, in the urine, was significant (Table 1).

Thus, the mechanism of action of oxytocin on the kidney, which is associated with an increase in sodium excretion and an increase in the reabsorption of solute-free water, can be explained on the basis of the classical concepts of the effect of neurohypophysial nonapeptides on the selective types of known receptors. Stimulation of oxytocin receptors in the proximal tubule reduces fluid reabsorption, which promotes saluresis, whereas the endogenous vasopressin secretion increases the reabsorption of solute-free water in the collecting ducts of the kidney.
